# Exogenous and Endogenous Serine Deficiency Exacerbates Hepatic Lipid Accumulation

**DOI:** 10.1155/2021/4232704

**Published:** 2021-10-19

**Authors:** Liuqin He, Yonghui Liu, Di Liu, Yanzhong Feng, Jie Yin, Xihong Zhou

**Affiliations:** ^1^Hunan Provincial Key Laboratory of Animal Intestinal Function and Regulation, Laboratory of Animal Nutrition and Human Health, College of Life Sciences, Hunan Normal University, Changsha 410081, China; ^2^Hunan Provincial Key Laboratory of Animal Nutritional Physiology and Metabolic Process, Institute of Subtropical Agriculture, Chinese Academy of Sciences, Changsha 410125, China; ^3^Institute of Animal Husbandry, Heilongjiang Academy of Agricultural Sciences, Harbin 150086, China; ^4^College of Animal Science and Technology, Hunan Agricultural University, Changsha 410128, China

## Abstract

Serine is involved in the regulation of hepatic lipid metabolism. However, whether exogenous or endogenous serine deficiency affects lipid accumulation in the liver and related mechanisms is unclear. Here, we investigated the effects of serine deficiency on hepatic fat accumulation in mice fed a serine-deficient diet or in mice supplemented with the D-3-phosphoglycerate dehydrogenase (PHGDH) inhibitor NCT-503. Both treatments produced an increase in body weight and liver weight and higher triglyceride content in the liver. Both treatments also exacerbated hepatic inflammatory responses and oxidative stress. Importantly, NCT-503 supplementation significantly inhibited PHGDH activity and decreased the serine content in the liver. Dietary serine deficiency significantly affected the colonic microbiota, characterized by a decreased ratio of *Firmicutes*/*Bacteroidetes* and decreased proportion of *Bifidobacterium*. Dietary serine deficiency additionally resulted in significantly decreased colonic and serum acetate and butyrate levels. The collective results indicate that NCT-503 supplementation may contribute to overaccumulation of hepatic lipid, by causing hepatic serine deficiency, while dietary serine deficiency may produce similar outcomes by affecting the gut-microbiota-liver axis.

## 1. Introduction

Hepatocytes are the most metabolically active cell type in the body, with diverse physiological and metabolic functions [1]. Decreased or increased metabolism of hepatocytes can result in complicated disorders and development of diseases, including fatty liver disease and cancer [2]. Imbalance in lipid metabolism in the liver is a hallmark of nonalcoholic fatty liver disease [3]. However, the underlying mechanisms leading to hepatic lipid overaccumulation and steatosis remain unclear.

Serine is a metabolically necessary amino acid that is a pivotal link between glycolysis and one-carbon and lipid metabolism, as well as purine and glutathione synthesis. Thus, serine has a critical role in a variety of biological functions. Genome-scale metabolic modeling of hepatocytes has demonstrated the involvement of serine deficiency in the development of fatty liver disease [1]. Importantly, exogenous serine supplementation can alleviate lipid overaccumulation and oxidative stress in the liver of subjects with high-fat-induced fatty liver disease or alcoholic fatty liver diseases [4, 5]. Interestingly, 3-phosphoglycerate dehydrogenase (PHGDH), the key enzyme involved in the de novo synthesis of serine, is also closely associated with the development of fatty liver diseases [6]. Knockdown of the *PHGDH* gene reduces hepatic serine content, while PHGDH-derived serine helps maintain general lipid homeostasis [7]. The aforementioned findings indicate that both exogenous and endogenous serine have critical roles in the regulation of hepatic lipid metabolism. However, whether exogenous serine deficiency affects hepatic lipid deposition remains unknown. Moreover, the possible effects of directly targeting the activity of PHGDH enzyme on hepatic lipid accumulation need to be explored.

Importantly, recent studies have focused on the effects of serine deficiency on inflammatory responses and oxidative stress [8, 9]. Whether exogenous or endogenous serine deficiency affects lipid accumulation in the liver and the related mechanisms remain to be elucidated. Subsequently, we conducted the current study to investigate the effects of exogenous serine deficiency by using a serine-deficient diet or endogenous serine deficiency by supplementing an PHGDH inhibitor NCT-503, on hepatic fat accumulation in mice. The results indicated that NCT-503 supplementation may contribute to overaccumulation of hepatic lipid, by causing hepatic serine deficiency, while dietary serine deficiency may produce similar outcomes by affecting the gut-microbiota-liver axis. Our results would enrich the understanding of serine as a modulator of lipid metabolism in liver and suggest the application of serine in lipid metabolism disorder-related diseases.

## 2. Materials and Methods

### 2.1. Animal Care and Experimental Design

Twenty-four C57BL/6 J male mice (6-week-old) were purchased from HUNAN Slac Laboratory Animal Central. All animals were housed in pathogen-free colonies at 22 ± 2°C, with a relative humidity of 50 ± 5% and a lighting cycle of 12 h/d. All animals had free access to food and water. All mice were randomly assigned into three treatment groups: (i) mice were fed on the control diet (CONT); (ii) mice were fed on the serine- and glycine-deficient diet (NS); and (iii) mice were fed on the control diet and supplemented with NCT-503 (NCT), a specific inhibitor of PHGDH. The experiment lasted six weeks. NCT-503 was supplemented with a daily dosage of 40 mg/kg body weight as previously did [10]. The diets were purchased from Research Diets (New Brunswick, NJ, USA), and diet composition was presented in Supplementary Table 1.

NCT-503 was purchased from Selleck (Shanghai, China).

The experimental protocol was approved by the Protocol Management and Review Committee of Institute of Subtropical Agriculture, and mice were treated according to the animal care guidelines of the Institute of Subtropical Agriculture (Changsha, China). During the experiment, body weight was recorded every week. At the end, the blood samples were obtained from the retroorbital sinus, and the serum was stored. Then, all mice were sacrificed by cervical dislocation, and the abdomen was opened to obtain and weigh the liver. Liver samples were collected and fixed in 4% formaldehyde for 24 h, and they were also collected and immediately frozen in liquid nitrogen. Colonic content was collected for the analysis of gut microbiota and short-chain fatty acids (SCFAs).

### 2.2. Biochemical Assays

Biochemical assays for aspartate aminotransferase (AST) and alanine aminotransferase (ALT) level were performed with commercially available kits (Beyotime, Shanghai, China).

### 2.3. Hematoxylin-Erosin and Oil Red O Staining

Hematoxylin-erosin (HE) and Oil Red O staining were performed as previously did [5]. Fresh liver samples immediately fixed in 4% formaldehyde, were paraffin embedded, sectioned into 8 *μ*m thickness, and then stained either with HE or Oil Red O.

### 2.4. Determination of Hepatic Triglycerides and Glutathione Contents

Hepatic lipid was extracted using a modified Folch method [11]. Triglycerides were measured using commercially available colorimetric kit (BSBE, Beijing, China). Reduced glutathione (GSH) content was measured using the corresponding commercial colorimetric assay kits (Beyotime, Shanghai, China) according to the manufacturer's instructions.

### 2.5. Determination of PHGDH Activity

PHGDH activity was determined using enzyme-linked immunosorbent assay according to the manufacturer's instructions (MEIMIAN, Suzhou, China). Protein concentration was determined by BCA Protein Assay (Pierce Biotechnology, Rockford, IL, USA).

### 2.6. RT-qPCR Analysis

RT-qPCR analysis was performed as previously did [12]. Total RNA was isolated from liver samples using TRIzol Reagent (Invitrogen), and cDNA was obtained using the PrimeScript RT reagent kit (Takara, Dalian, China). RT-qPCR was performed using SYBR Green mix (Takara). All samples were run in triplicate, and the results were calculated by normalizing the mRNA expression of target genes to *β*-actin mRNA. The primer sequences [13] are shown in Supplementary Table 2.

### 2.7. Determination of Reactive Oxygen Species Content

Hepatic reactive oxygen species (ROS) content was determined as previously did [14] . As abovementioned, 10 *μ*m sections were stained with dihydroethidium (Sigma-Aldrich) for 20 min at 37°C in a humidified 5% CO_2_ incubator. Representative pictures were captured by fluorescence microscopy, and fluorescence intensity was calculated by Image Browser software (Leica, Wetzlar, Germany).

### 2.8. Determination of Serine Concentration

Seine concentration in serum and liver was measured as previously did [15]. Briefly, grounded liver samples (50 mg) and serum were added with 10% sulfosalicylic acid. After completely vortexed, the supernatant was obtained after centrifuged at 12 000 g for 10 minutes and filtered through 0.22 *μ*m filters for the determination of serine concentration.

### 2.9. Gut Microbiota Profiling

Gut microbiota profiling was assayed as previously described [8]. Briefly, DNA was extracted from colonic contents and isolated using the QIAamp DNA stool Mini Kit (Qiagen, Shanghai, China). Bacterial 16S rDNA gene sequences (V3–V4 region) were amplified, and PCR was performed using Phusion High-Fidelity PCR Master Mix reagent (New England BioLabs Inc). Amplicons purified using Qiagen Gel Extraction Kit (Qiagen) were sequenced using the Illumina HiSeq2500 platform. Quality filtering and analysis were performed using USEARCH, while adhering to the QIIME quality-controlled process based on 97% sequence similarity (Novogene, Beijing, China).

### 2.10. Determination of SCFA Content

SCFAs were measured as previously did [16]. Briefly, fecal samples were collected and ground in liquid nitrogen. Then, the samples were mixed with pure water (300%, w/v), homogenized and centrifuged to collect fecal homogenate. Serum samples were collected after centrifugation at 1200 g for 15 min at 4°C. Then, the fecal homogenate or serum was mixed with 5 M HCl and extracted with anhydrous diethyl ether. Next, the extracts were derivatized with O-bis(trimethyl-silyl)-trifuoroacetamide. Finally, the profiling of SCFAs was analyzed by the gas chromatography/mass spectrometry.

### 2.11. Statistical Analysis

Significance between treatments was analyzed using one-way ANOVA followed by Student–Newman–Keuls post hoc test, using the data statistics software SPSS 18.0. Data are presented as means ± SEM. Mean values were considered significantly different when *P* < 0.05.

## 3. Results

### 3.1. Serine Deficiency Resulted in Increased Body Weight Gain and Impaired Liver Morphology

**Figure 1:**
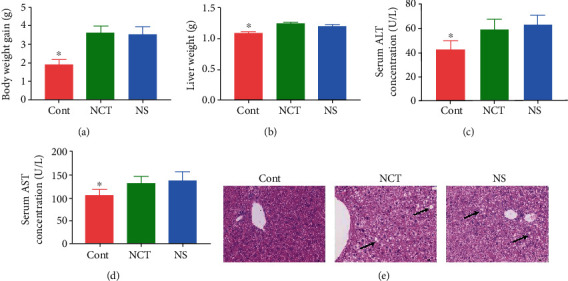
Serine deficiency resulted in increased body weight gain and impaired liver morphology: (a) body weight gain, (b) liver weight, ALT (c) and AST (d) concentrations in serum, and (e) HE staining (×200). Arrows, impaired hepatocytes. CONT: mice were fed on the control diet; NS: mice were fed on the serine-and glycine-deficient diet; NCT: mice were fed on the control diet and supplemented with NCT-503. ALT: alanine aminotransferase; AST: aspartate aminotransferase. Data are presented as means ± SEM. *n* = 8. ^∗^Mean values were significantly different between CONT and NCT, NS (*P* < 0.05).

As shown in Figure 1, body weight gain and liver weight were significantly lower in the CONT group than those in the NCT and NS group (Figures 1(a) and 1(b)). To investigate the effects of serine deficiency on the liver function, we firstly determined serum concentrations of ALT and AST. The results showed that ALT and AST concentrations were significantly lower in the CONT group than those in the NCT and NS group (Figures 1(c) and 1(d)). Additionally, the results of HE staining showed that the liver morphology was impaired in both the NCT and NS group, as indicated by vacuolization and damaged hepatocytes (Figure 1(e)).

### 3.2. Serine Deficiency Exacerbated Hepatic Lipid Accumulation

**Figure 2:**
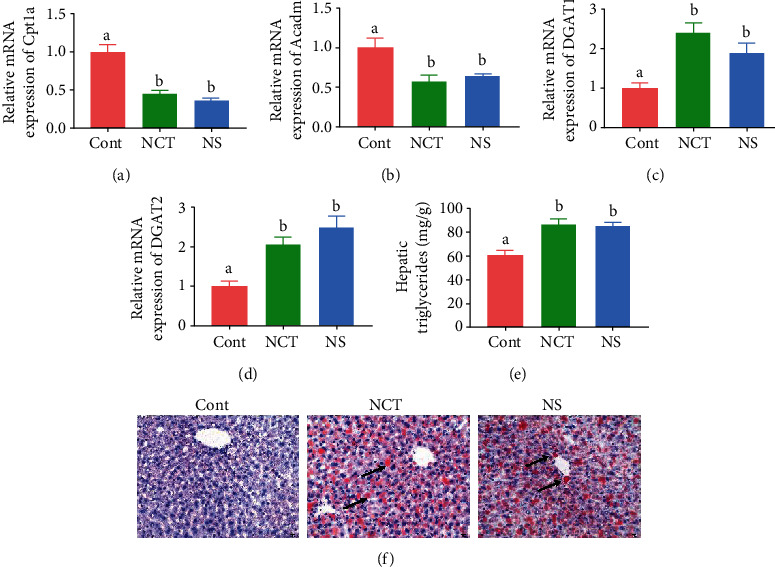
Serine deficiency exacerbated hepatic lipid accumulation. Cpt1a: carnitine palmitoyltransferase 1a; Acadm: medium-chain acyl-CoA dehydrogenase; DGAT: diacylglycerol O-acyltransferase. Relative mRNA expression of *Cpt1a* (a), *Acadm* (b), *DGAT1* (c), and *DGAT2* (d). (e) Oil Red O staining (×200). Arrows, lipid droplet. CONT: mice were fed on the control diet; NS: mice were fed on the serine- and glycine-deficient diet; NCT: mice were fed on the control diet and supplemented with NCT-503. Data are presented as means ± SEM. *n* = 8. ^a,b^Means of the bars with different letters were significantly different among groups (*P* < 0.05).

To investigate whether serine deficiency affects lipid metabolism in the liver, we determined the expression of genes involved in fatty acid oxidation and triglyceride synthesis. The results showed that the mRNA expression of *Cpt1a* and *Acadm* was significantly decreased (Figures 2(a) and 2(b)) while the mRNA expression of *DGAT1* and *DGAT2* was significantly increased (Figures 2(c) and 2(d)) in the liver of mice in the NCT and NS group, when compared with those of the CONT group. Furthermore, hepatic triglyceride content was significantly higher in mice in the NCT and NS group than those in the CONT group (Figure 2(e)). The results of Oil Red O staining further confirmed an increased accumulation of lipid in mice in the NCT and NS group (Figure 2(f)).

### 3.3. Serine Deficiency Aggravated Oxidative and Inflammatory Status in the Liver

**Figure 3:**
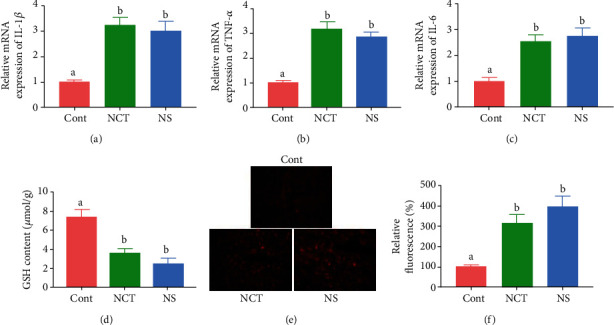
Serine deficiency aggravated oxidative and inflammatory status in liver. GSH: glutathione. Relative mRNA expression of *IL-1β* (a), *TNF-α* (b), and *IL-6* (c). (d) GSH content in the liver. (e) ROS content (red) in the liver. (f) Relative fluorescence of ROS staining. CONT: mice were fed on the control diet; NS: mice were fed on the serine- and glycine-deficient diet; NCT: mice were fed on the control diet and supplemented with NCT-503. Data are presented as means ± SEM. *n* = 8 for the mRNA expression and GSH content and *n* = 3 for ROS staining. ^a,b^Means of the bars with different letters were significantly different among groups (*P* < 0.05).

To investigate whether serine deficiency affects inflammatory responses, we determined genes expression of inflammatory cytokines in the liver. The results showed that the mRNA expression of *IL-1β, TNF-α*, and *IL-6* was significantly increased in mice in the NCT and NS group, when compared with those of the CONT group (Figures 3(a) and 3(c)). To investigate whether serine deficiency affects oxidative status, we determined the content of GSH and ROS in the liver. The results showed that GSH content was significantly decreased (Figure 3(d)) while ROS level was significantly increased (Figures 3(e) and 3(f)) in mice in the NCT and NS group, when compared with those of the CONT group.

### 3.4. Dietary NCT-503 Supplementation Decreased Serine Content in the Liver

**Figure 4:**
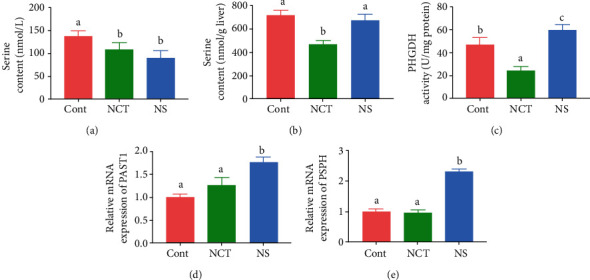
Dietary NCT-503 supplementation decreased serine content in liver. PHGDH: phosphoglycerate dehydrogenase; PAST1: phosphoserine aminotransferase; PSPH: phosphoserine phosphatase. Serine content in serum (a) and liver (b). (c) PHGDH activity. Relative mRNA expression of *PAST1* (d) and *PSPH* (e). CONT: mice were fed on the control diet; NS: mice were fed on the serine-and glycine-deficient diet; NCT: mice were fed on the control diet and supplemented with NCT-503. Data are presented as means ± SEM. *n* = 8. ^a,b,c^Means of the bars with different letters were significantly different among groups (*P* < 0.05).

To investigate whether serine deficiency affects serine content in mice, we determined serine content in the serum and liver. The results showed that serine content in serum was significantly decreased in mice in the NCT and NS group, when compared with those of the CONT group (Figure 4(a)). Notably, serine content in the liver was significantly decreased in mice in the NCT group, while it was not significantly changed in mice in the NS group (Figure 4(b)). Importantly, PHGDH activity (Figure 4(c)) and mRNA expression of *PAST1* and *PSPH* were significantly higher in mice in the NS group (Figures 4(d) and 4(e)), while PHGDH activity was significantly lower in mice in the NCT group (Figure 4(c)), when compared with those of the CONT group.

### 3.5. Dietary Serine Deficiency Altered Microbiota Composition in Colonic Content

**Figure 5:**
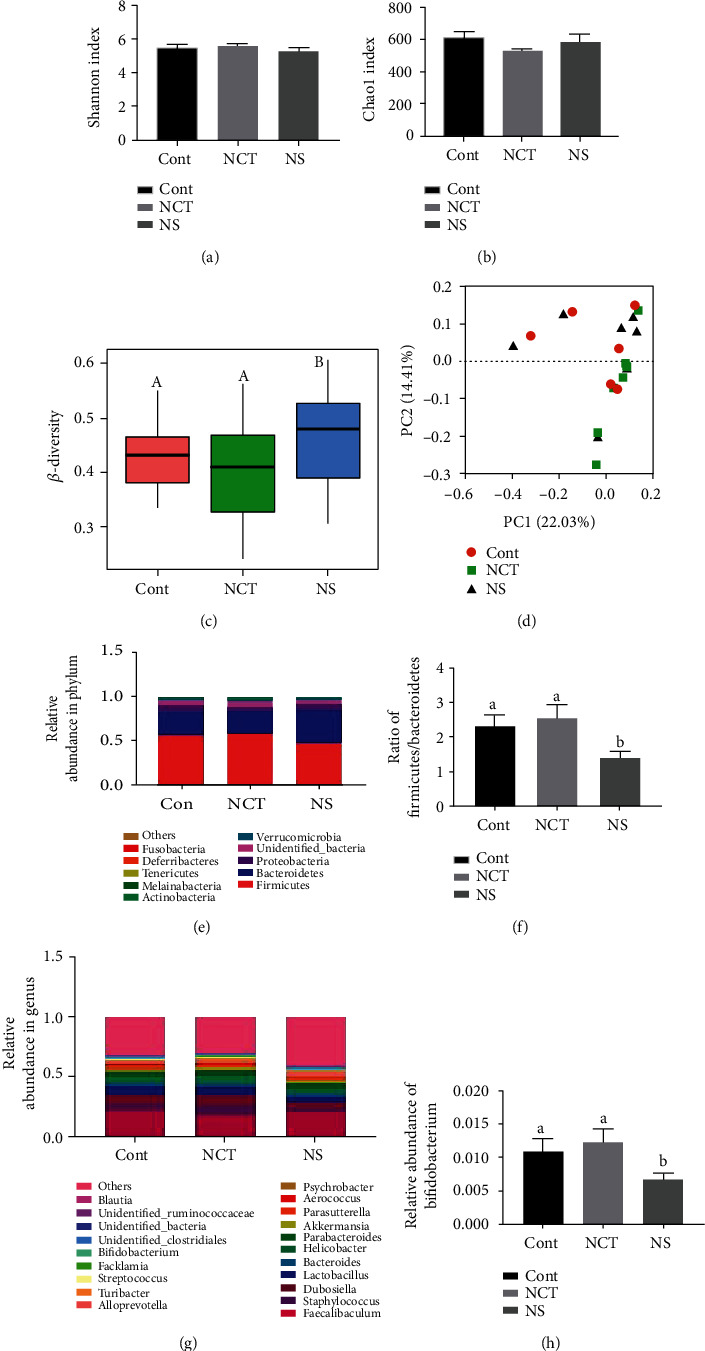
Dietary serine deficiency altered microbiota composition in colonic content. (a) Shannon index. (b) Chao1 index. (c) *β*-Diversity. (d) PCoA plot of the microbiota based on an unweighted UniFrac metric. (e) Relative abundance of predominant bacteria at the phylum level. (f) Ratio of *Firmicutes* to *Bacteroidetes*. (g) Relative abundance of predominant bacteria at the genus level. (h) Relative abundance of *Bifidobacterium*. CONT: mice were fed on the control diet; NS: mice were fed on the serine- and glycine-deficient diet; NCT: mice were fed on the control diet and supplemented with NCT-503. Data are presented as means ± SEM. *n* = 6 for the CONT group and *n* = 7 for the NCT and NS group. ^a,b^Means of the bars with different letters were significantly different among groups (*P* < 0.05). ^A,B^Means of the bars with different letters were tended to differ between NS and CONT and NCT (0.05 < *P* < 0.1, which was considered as a tendency).

To investigate whether serine deficiency altered microbiota composition, we used 16S rDNA gene sequencing for bacterial identification in colonic content. The results showed that no significant change was observed in microbiota composition between mice in the CONT group and NCT group (Figure 5). Although no significant changes were observed in Shannon and Chao1 index (Figures 5(a) and 5(b)), *β*-diversity was tended to increase (Figure 5(c)) in mice in the NS group, when compared with those of the CONT group. Unweighted principal coordinate analysis indicated no clear difference in beta-diversity among the treatment groups (Figure 5(d)). Notably, the ratio of *Firmicutes* to *Bacteroidetes* (Figures 5(e) and 5(f)) and the relative abundance of *Bifidobacterium* (Figures 5(g) and 5(h)) were decreased in mice in the NS group, when compared with those of the CONT group.

### 3.6. Dietary Serine Deficiency Decreased Contents of SCFAs in Colonic Contents and Serum

**Figure 6:**
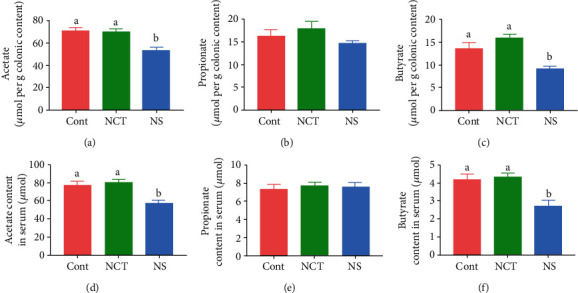
Dietary serine deficiency decreased contents of short-chain fatty acids in colonic contents and serum. Acetate (a), propionate (b), and butyrate (c) content in colonic content. Acetate (d), propionate (e), and butyrate (f) content in serum. CONT: mice were fed on the control diet; NS: mice were fed on the serine- and glycine-deficient diet; NCT: mice were fed on the control diet and supplemented with NCT-503. Data are presented as means ± SEM. *n* = 8. ^a,b^Means of the bars with different letters were significantly different among groups (*P* < 0.05).

To further investigate whether the changes of microbiota composition accompanied by changes of metabolites, we determined the contents of SCFAs. As shown in Figure 6, dietary serine deficiency significantly decreased acetate and butyrate contents in both colonic contents and serum in mice, while it had no effects on propionate content. Additionally, NCT-503 supplementation had no effects on acetate, propionate, and butyrate contents in either colonic contents or serum in mice.

## 4. Discussion

**Figure 7:**
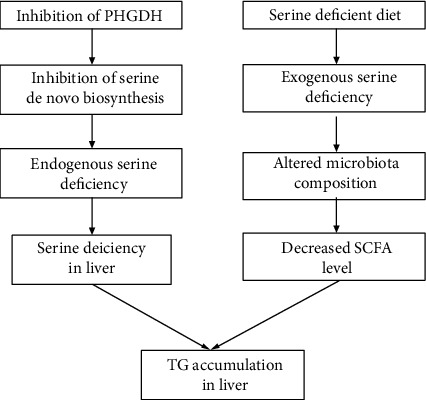
The different mechanisms responsible for hepatic lipid accumulation caused by endogenous and exogenous serine deficiency.

Both exogenous and endogenous serine are involved in the regulation of lipid metabolism [1, 6]. In the present study, we found that both endogenous serine deficiency via inhibition of de novo serine synthesis and exogenous serine deficiency resulted in increases in body weight and liver weight. Increased liver weight was accompanied by increased lipid accumulation. Inhibition of PHGDH activity resulted in decreased serine content in the liver, which may contribute to aggravated oxidative and inflammatory status, whereas dietary serine deficiency did not affect the serine content in the liver. Notably, dietary serine deficiency decreased the ratio of *Firmicutes* to *Bacteroidetes* in the colonic microbiota. Importantly, dietary serine deficiency also decreased the relative colonic abundance of *Bifidobacterium* and the metabolites of acetate and butyrate. These changes may explain why exogenous serine deficiency results in lipid overaccumulation in the liver. Our results suggest that exogenous and endogenous serine deficiencies disrupt hepatic lipid metabolism by different mechanisms (Figure 7).

Serine can be derived from food and protein turnover and can be synthesized de novo from 3-phosphoglycerate and directly from glycine. Of these mechanisms, de novo biosynthesis is the main mechanism. As expected, dietary serine deficiency and inhibition of PHGDH caused a significant decrease in serum serine content in mice. Notably, only inhibition of PHGDH caused reduction in the liver serine content. Hepatic serine content was not affected by dietary serine deficiency. Surprisingly, we observed increased hepatic PHGDH activity and upregulated expression of *PAST1* and *PSPH*, which encode the enzymes involved in de novo serine biosynthesis. These results indicate that the increase in de novo biosynthesis may compensate for dietary serine deficiency to meet the serine requirement in the liver.

Hepatic serine is closely involved in lipid metabolism [4, 5, 7]. Hepatic serine deficiency contributes to the overaccumulation of lipids in the liver [1, 6]. In the present study, we further confirmed that the inhibition of de novo serine biosynthesis by administration of NCT-503 resulted in hepatic serine deficiency and then lipid accumulation, as indicated by the results of Oil Red O staining and increased hepatic triglyceride content. A previous study had shown that a lack of the *PHGDH* gene expression may inhibit SIRT1 activity and further increase lipid accumulation [6]. However, in the present study, we directly targeted PHGDH activity, which may have produced different responses. Consequently, the underlying mechanisms by which serine deficiency in the liver contributes to lipid accumulation need to be studied further. Disturbances in lipid metabolism are often accompanied by increased inflammatory responses and oxidative stress. We found increased expression of proinflammatory cytokines, decreased GSH content, and increased ROS content in the liver of mice treated with NCT-503. These results suggest that inhibition of de novo serine biosynthesis exacerbates the inflammatory and oxidative status in the liver.

Dietary serine deficiency did not cause changes in hepatic serine content. Thus, other factors may be involved in the disturbance of lipid accumulation and oxidative and inflammatory status. Dysbiosis in the gut microbiota contributes to the pathogenesis of fatty liver disease [17]. Changes in the microbiome and their metabolites can result in liver inflammation and fibrosis. We previously described that either dietary serine supplementation or serine deficiency can regulate gut microbiota composition [8, 18]. These observations suggest that dietary serine deficiency may affect hepatic lipid accumulation through the gut-microbiota-liver axis. Importantly, the ratio of *Firmicutes* to *Bacteroidetes* and the relative abundance of *Bifidobacterium* decreased in mice fed a serine deficiency diet. Although the association of *Firmicutes* and *Bacteroidetes* abundance with fatty liver disease remains to be contradictory [19, 20], an important function of the *Bifidobacterium* genus is to produce acetate, which can be converted into butyrate by other bacteria [21, 22]. These results suggest that dietary serine deficiency may affect the gut microbiota and thus cause changes in their metabolites, including short-chain fatty acids. We also observed decreased contents of acetate and butyrate in both the serum and colonic contents. It has been suggested that SCFAs can ameliorate lipogenesis, inflammatory response, and oxidative damage in the liver tissue [23]. We hypothesize that the decreased production of SCFAs affected by dietary serine deficiency may contribute to lipid overaccumulation in the liver. However, whether decreased *Bifidobacterium* genus contributes to the decreased SCFAs and then results in fatty liver needs to be studied.

## 5. Conclusions

In the present study, exogenous and endogenous serine deficiencies exacerbated hepatic lipid accumulation via different mechanisms. Specifically, inhibition of serine de novo biosynthesis resulted in hepatic serine deficiency and further contributed to the fatty liver, while dietary serine deficiency altered the microbiota composition and further resulted in the development of fatty liver via the gut-microbiota-liver axis.

## Data Availability

The data used to support the findings of this study are available from the corresponding author upon request.

## Supplementary Materials

Supplementary Materials Supplementary Table 1: components of the control diet, serine, and glycine deficient (SGD) diet used in the experiments. These two diets were purchased from Research Diets (New Brunswick, NJ, USA). Supplementary Table 2: sequences of primers used for RT-qPCR in the experiments. *Cpt1a*: carnitine palmitoyltransferase 1a; *Acadm*: medium-chain acyl-CoA dehydrogenase; *DGAT*: diacylglycerol O-acyltransferase; *PAST1*: phosphoserine aminotransferase; *PSPH*: phosphoserine phosphatase.
