# Supplementary material for: Exogenous and Endogenous Serine Deficiency Exacerbates Hepatic Lipid Accumulation
Source: Oxid Med Cell Longev. 2021 Oct 19;2021:4232704. doi: 10.1155/2021/4232704 (PMC8548146; doi:10.1155/2021/4232704)
Supplement: Supplementary Materials — Supplementary Table 1: components of the control diet, serine, and glycine deficient (SGD) diet used in the experiments. These two diets were purchased from Research Diets (New Brunswick, NJ, USA). Supplementary Table 2: sequences of primers used for RT-qPCR in the experiments. Cpt1a: carnitine palmitoyltransferase 1a; Acadm: medium-chain acyl-CoA dehydrogenase; DGAT: diacylglycerol O-acyltransferase; PAST1: phosphoserine aminotransferase; PSPH: phosphoserine phosphatase. [file 4232704.f1.docx]

**Supplementary table 1** Components of the control diet, and serine and glycine deficient (SGD) diet used in the experiments. These two diets were purchased from Research Diets (New Brunswick, NJ, USA).

**Supplementary table 2** Sequences of primers used for RT-qPCR in the experiments.

**Supplementary table 1** Diet components.

| Components | Control diet, gm | SGD diet, gm |
| --- | --- | --- |
| L-Arginine | 10 | 10 |
| L-Histidine-HCl-H_2_O | 6 | 6 |
| L-Isoleucine | 8 | 8 |
| L-Leucine | 12 | 12 |
| L-Lysine-HCl | 14 | 14 |
| L-Methionine | 6 | 6 |
| L-Phenylalanine | 8 | 8 |
| L-Threonine | 8 | 8 |
| L-Tryptophan | 2 | 2 |
| L-Valine | 8 | 8 |
| L-Alanine | 10 | 10 |
| L-Asparagine-H2O | 5 | 5 |
| L-Aspartate | 10 | 10 |
| L-Cystine | 4 | 4 |
| L-Glutamic Acid | 30 | 30 |
| L-Glutamine | 5 | 5 |
| L-Glycine | 10 | 0 |
| L-Proline | 5 | 5 |
| L-Serine | 5 | 0 |
| L-Tyrosine | 4 | 4 |
| Corn Starch | 550.5 | 565.5 |
| Maltodextrin 10 | 125 | 125 |
| Cellulose | 50 | 50 |
| Corn Oil | 50 | 50 |
| Mineral Mix S10001 | 35 | 35 |
| Sodium Bicarbonate | 7.5 | 7.5 |
| Vitamin Mix V10001 | 10 | 10 |
| Choline Bitrartrate | 2 | 2 |
| Total | 1000 | 1000 |

**Supplemental Table 2 Primers for RT-qPCR analysis**

| Gene | 5’-3’ Primer sequence |  |
| --- | --- | --- |
| *Cpt1a* | F: CAGTCGACTCACCTTTCCTG | |
|  | R: CATCATGGCTTGTCTCAAGTG | |
| *Acadm* | F: TGCTCGCAGAAATGGCGATGA | |
|  | R: CAATGTGCTCACGAGCTATGA | |
| *DGAT1* | F: TTCCGCCTCTGGGCATT | |
|  | R: AGAATCGGCCCACAATCCA | |
| *DGAT2* | F: AGTGGCAATGCTATCATCATCGT | |
|  | R: TCTTCTGGACCCATCGGCCCCAGGA | |
| *IL-1β* | F: TGCCACCTTTTGACAGTGATG | |
|  | R: AAGGTCCACGGGAAAGACAC | |
| *TNF-α* | F: ATGAGAAGTTCCCAAATGGC | |
|  | R: CTCCACTTGGTGGTTTGCTA | |
| *IL-6* | F: CCTCTCTGCAAGAGACTTCCAT | |
|  | R: AGTCTCCTCTCCGGACTTGT | |
| *PAST1* | F: ACGCCAAAGGAGACGAAGCT | |
|  | R: ATGTTGAGTTCTACCGCCTTGTC | |
| *PSPH* | F: TAAGGTTCCGTTGTGCTCGC | |
|  | R: GAAGCATCCCTCACACACGA | |
| *β-actin* | F: TGTCCACCTTCCAGCAGATGT | |
|  | R: AGCTCAGTAACAGTCCGCCTAGA | |
